# Supplementary figures and images for: An image J plugin for the high throughput image analysis of in vitro scratch wound healing assays
Source: PLoS One. 2020 Jul 28;15(7):e0232565. doi: 10.1371/journal.pone.0232565 (PMC7386569; doi:10.1371/journal.pone.0232565)

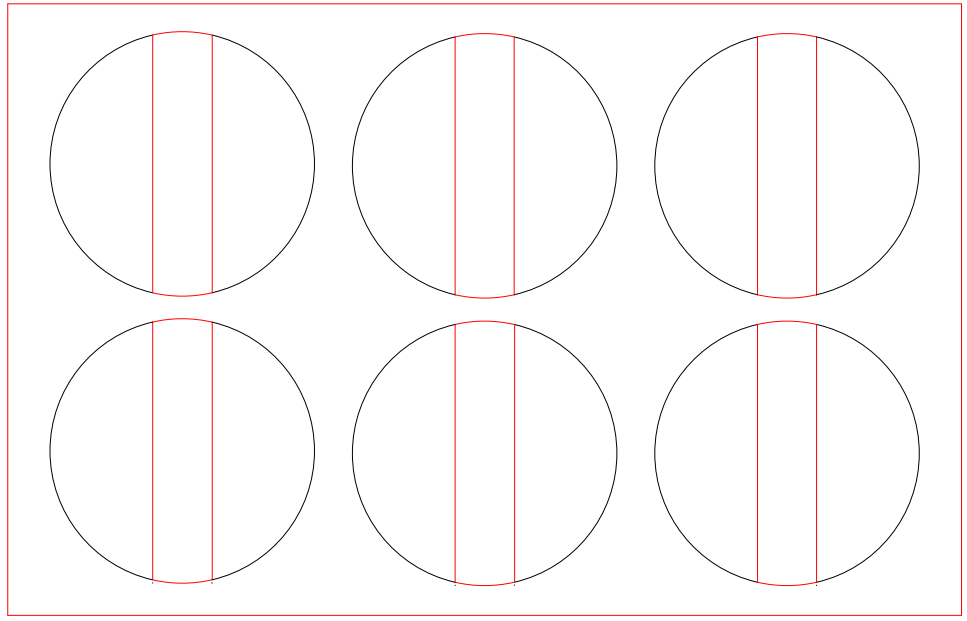

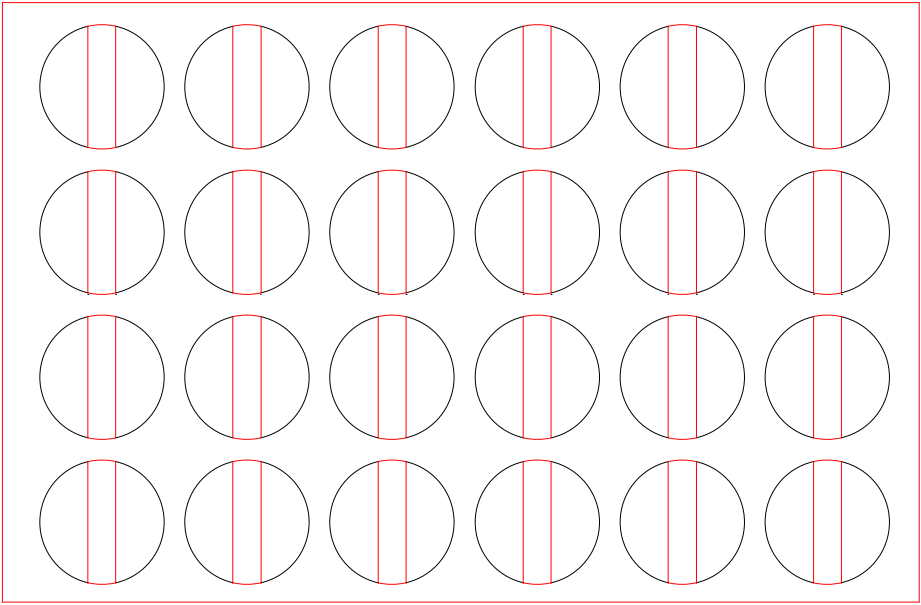

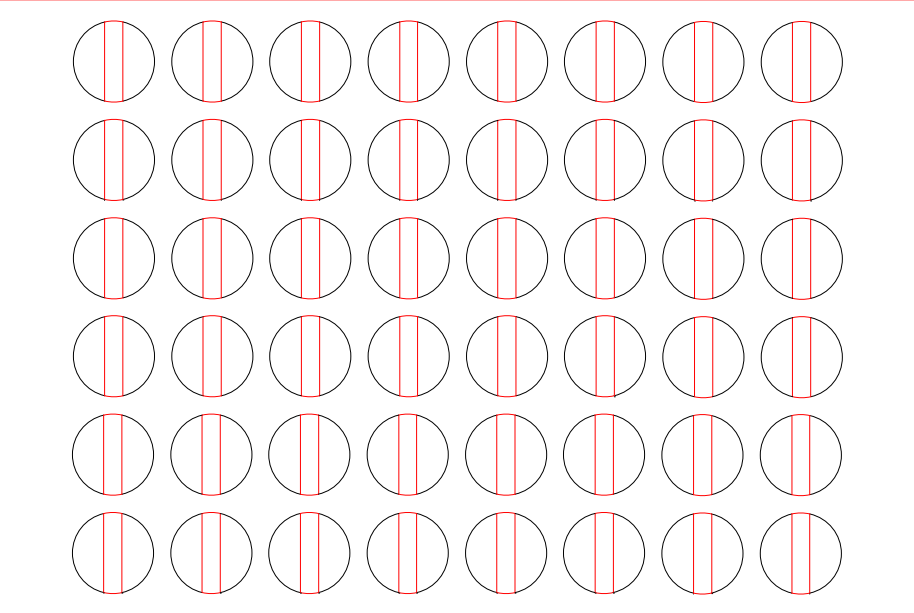

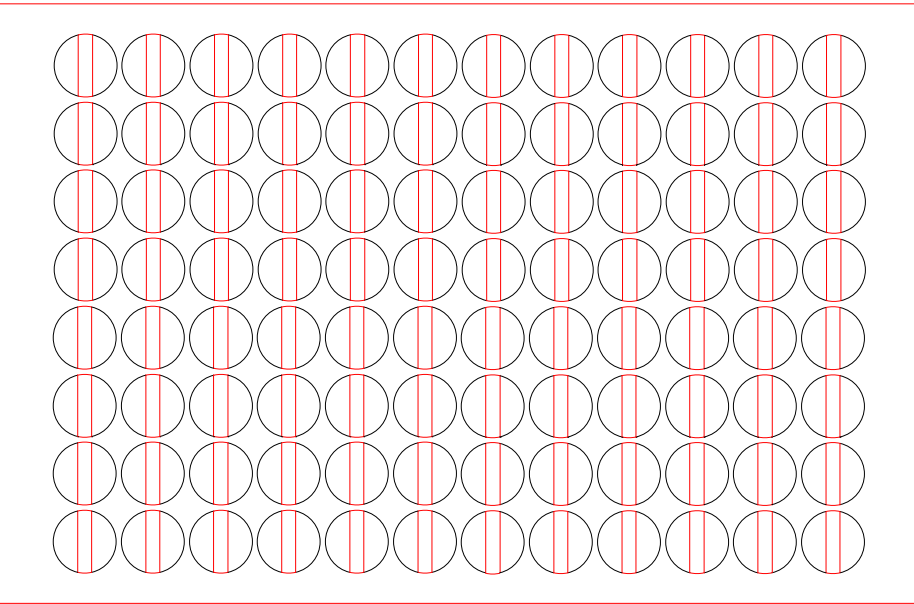

Supplement: S1 File — (PDF) [file pone.0232565.s001.pdf]

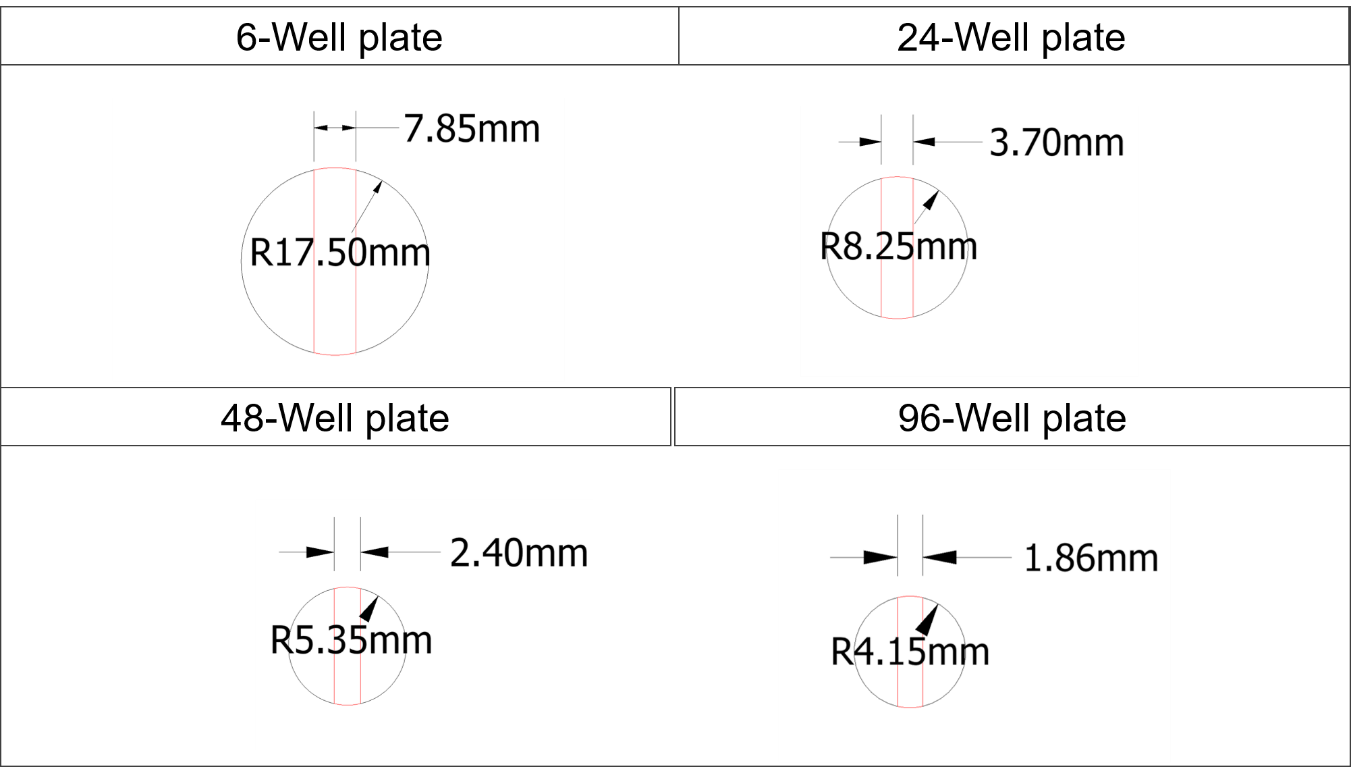


**S1 Fig.** Schemes for the Wound formation molds for 6, 24, 48 and 96-well plates

Supplement: S1 Fig — (DOCX) [file pone.0232565.s003.docx]
