# Supplementary material for: An image J plugin for the high throughput image analysis of in vitro scratch wound healing assays
Source: PLoS One. 2020 Jul 28;15(7):e0232565. doi: 10.1371/journal.pone.0232565 (PMC7386569; doi:10.1371/journal.pone.0232565)
Supplement: S2 File — (DOCX) [file pone.0232565.s002.docx]

# Supplementary information 2

## Manual Wound_healing_size_tool (Individual image analysis)

### Image acquisition and plugin preparation

- Download in <https://github.com/AlejandraArnedo/Wound-healing-size-tool/wiki> and unzip the Wound_healing_size_tool.ijm.
- Acquire the time-lapse images (∗ .tif, ∗ .oif, ∗ .zvi or equivalent). or single images (*.tif, *jpg, *.png or equivalent.
- Start the Open-source software (ImageJ/Fiji), and open the images by clicking *File → Open.*
- If you have images with multiple channels clicking *Image → Color → Split channels* and select the channel to analyze.
- If you have a stack clickling *Image→ Stacks→ Stack to images* and select the image to analyze.
- If you have an image in pixel calibrate the image scale clicking *Analyze → Set scale* and introduce the correct scale (pixels/µm).

### Wound_healing_size_tool

- Install the plugin clicking *Puglins→Macros→Install...* and select the file *Wound_healing_size_tool.ijm.*
- If the scratch is horizontal rotate the image clicking in the icon
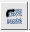

- Perform the plugin clicking in the icon
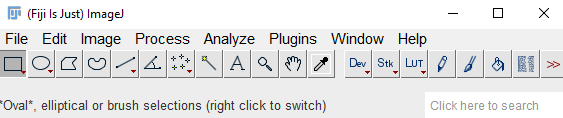
, set the value of the parameters and select the image.


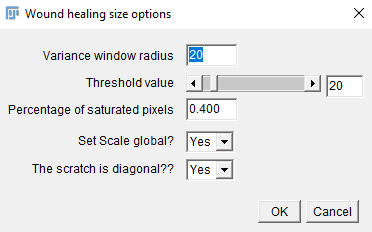


- - Variance window radius: Represent the radius of variance filter which is established to determine the empty or the occupied zones. The radius must be big enough, so the noise variance has no impact on tissue variance.
  - Threshold: The image resulting from the variance filter is converted to a mask by applying the given threshold.
  - Percentages of saturated pixels: Enhance the contrast of the image by determining the number of pixels in the image that can become saturated. Increasing this value increases contrast. This value should be greater than zero.
  - Set Scale global? Select “Yes” if the scale calibration applies to all the images. Select “No” if the scale calibration only applies to the select image.
  - The scratch is diagonal? Select “Yes” if you can observe that the scratch has an inclination differently of 0°. Select “No” if the scratch has not an inclination.
- Finalize clicking in the button OK.
- Verify if the select ROi is the wound area.


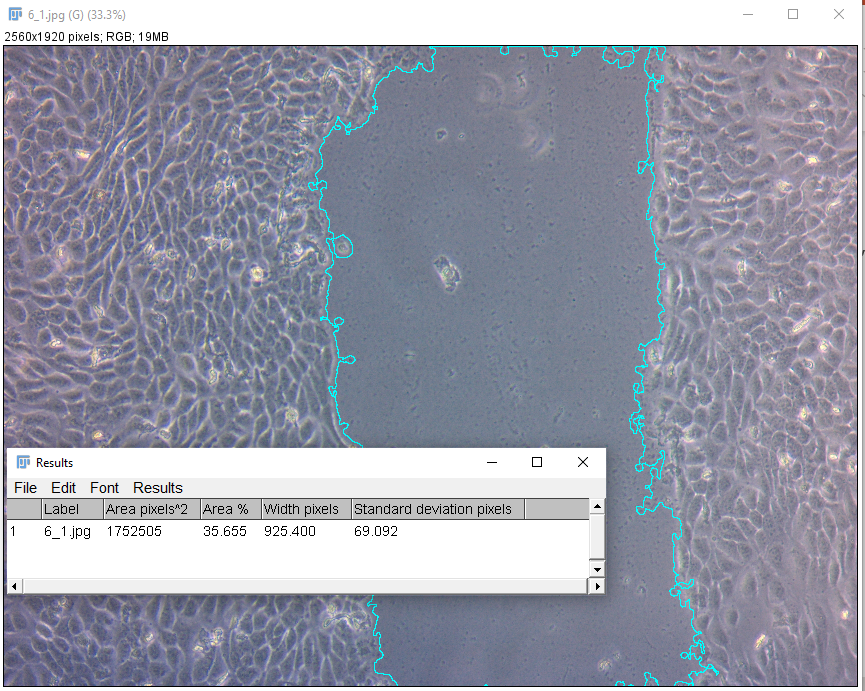


- Repeat the procedure with all the images.
- Copy the table results and analyze the data.

## Manual Wound_healing_size_tool (Stack analysis)

### Image acquisition and preparation

- Acquire the time-lapse images (∗ .tif, ∗ .oif, ∗ .zvi or equivalent). or single images (*.tif, *jpg, *.png or equivalent.
- Start the Open-source software (ImageJ/Fiji), and open the images by clicking *File → Open.*
- If you have an image in pixel calibrate the image scale clicking *Analyze → Set scale* and introduce the correct scale (pixels/µm).

### Wound_healing_size_tool

- Install the plugin clicking *Puglins→Macros→Install...* and select the file *Wound_healing_size_tool.ijm.*
- If the scratch is horizontal rotate the image clicking in the icon
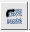

- Perform the plugin clicking in the icon
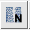
, set the value of the parameters and select the stack:


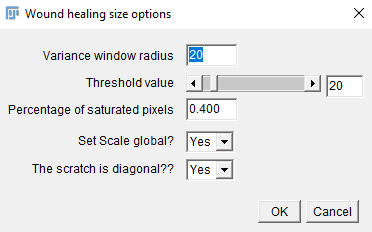


- - Variance window radius: Represent the radius of variance filter which is established to determine the empty or the occupied zones. The radius must be big enough, so the noise variance has no impact on tissue variance.

Threshold: The image resulting from the variance filter is converted to a mask by applying the given threshold.

- - Percentages of saturated pixels: Enhance the contrast of the image by determining the number of pixels in the image that can become saturated. Increasing this value increases contrast. This value should be greater than zero.
  - Set Scale global? Select “Yes” if the scale calibration applies to all the images. Select “No” if the scale calibration only applies to the select image.
  - The scratch is diagonal? Select “Yes” if you can observe that the scratch has an inclination differently of 0°. Select “No” if the scratch has not an inclination.
- Finalize clicking in the button OK.
- Wait until the macro run and pops up the Results window
- Copy all the results and analyze the data.

**Hint:** Before performing the analysis of the stack verify the parameters doing an individual image analysis with one duplicate image of the stack.
