# Supplementary material for: An image J plugin for the high throughput image analysis of in vitro scratch wound healing assays
Source: PLoS One. 2020 Jul 28;15(7):e0232565. doi: 10.1371/journal.pone.0232565 (PMC7386569; doi:10.1371/journal.pone.0232565)
Supplement: S2 Fig — (DOCX) [file pone.0232565.s004.docx]

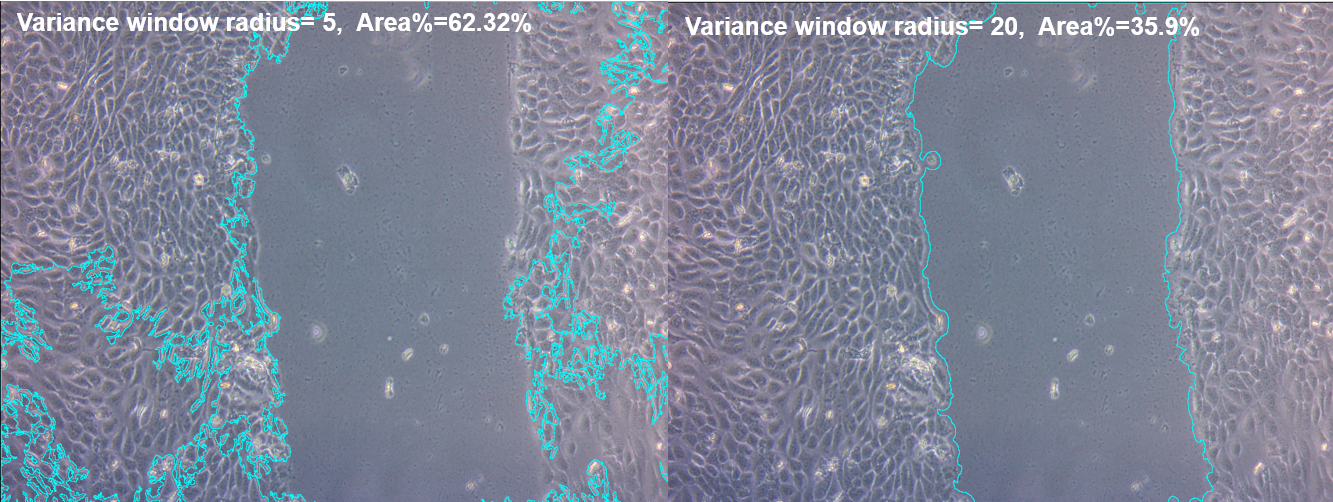


**S2 Fig.** Wound healing size test processed with two different values of variance window radius on the same image**.**
