# Supplementary material for: An image J plugin for the high throughput image analysis of in vitro scratch wound healing assays
Source: PLoS One. 2020 Jul 28;15(7):e0232565. doi: 10.1371/journal.pone.0232565 (PMC7386569; doi:10.1371/journal.pone.0232565)
Supplement: S3 Fig — A. Effect of changing the variance window radius while maintaining constant the threshold value = 100 and the percentage of saturated pixels = 0.01. B. Effect of changing the threshold value while maintaining constant the variance window radius = 20 and the percentage of saturated pixels = 0.01. C. Effect of changing the percentage of saturated pixels while maintaining constant the variance window radius = 20 and the threshold value = 100. (DOCX) [file pone.0232565.s005.docx]

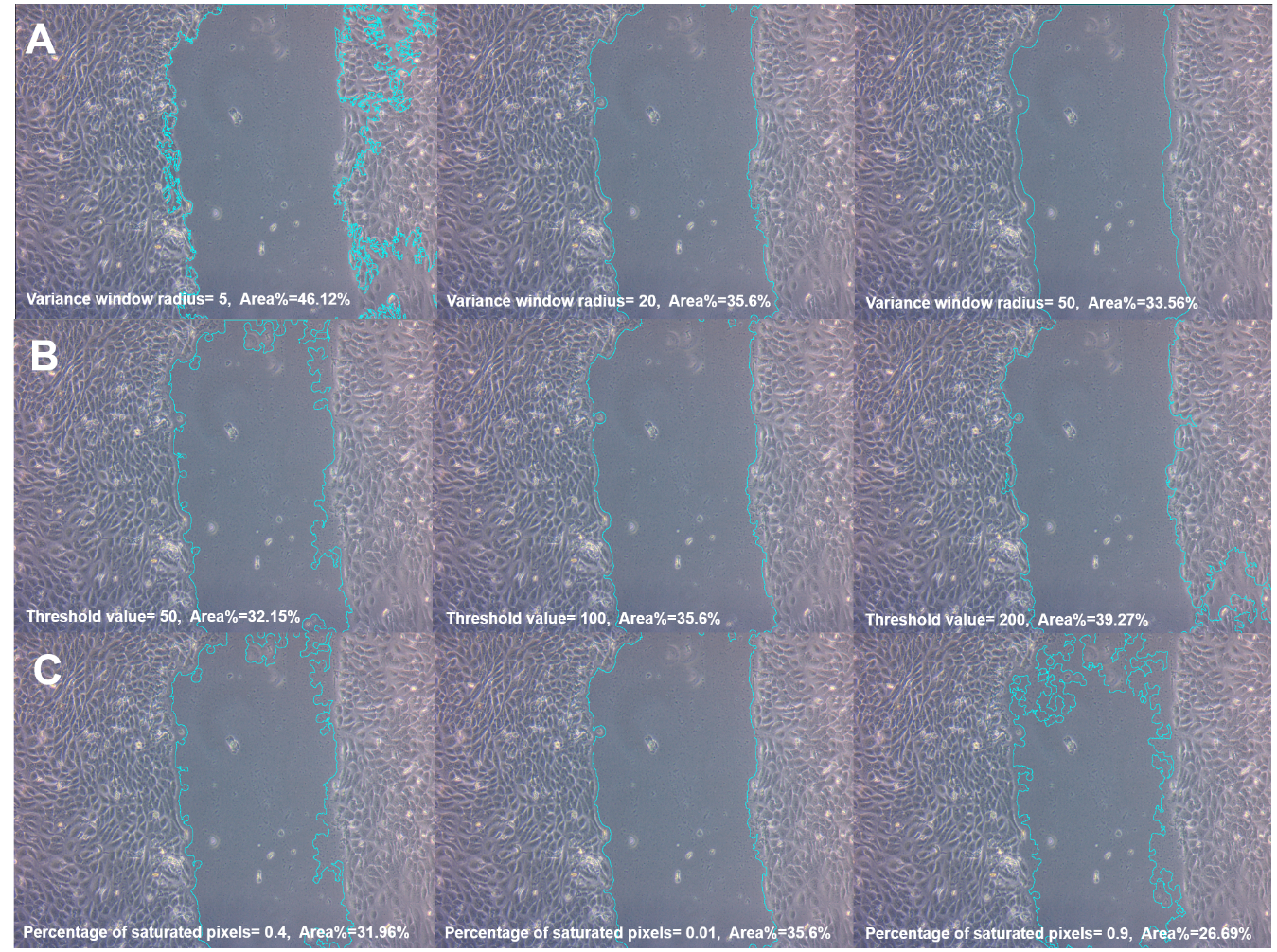


**S3 Fig. Wound healing size parameter selection effect on the detected area. A**. Effect of changing the variance window radius while maintaining constant the threshold value =100 and the percentage of saturated pixels = 0.01. **B**. Effect of changing the threshold value while maintaining constant the variance window radius= 20 and the percentage of saturated pixels = 0.01. **C.** Effect of changing the percentage of saturated pixels while maintaining constant the variance window radius= 20 and the threshold value =100.
