# Supplementary material for: An image J plugin for the high throughput image analysis of in vitro scratch wound healing assays
Source: PLoS One. 2020 Jul 28;15(7):e0232565. doi: 10.1371/journal.pone.0232565 (PMC7386569; doi:10.1371/journal.pone.0232565)
Supplement: S4 Fig — A. Wounds with different angles of inclination. Scale bar = 200 pixels. B. Width measurements with (blue) and without correction (white). (DOCX) [file pone.0232565.s006.docx]

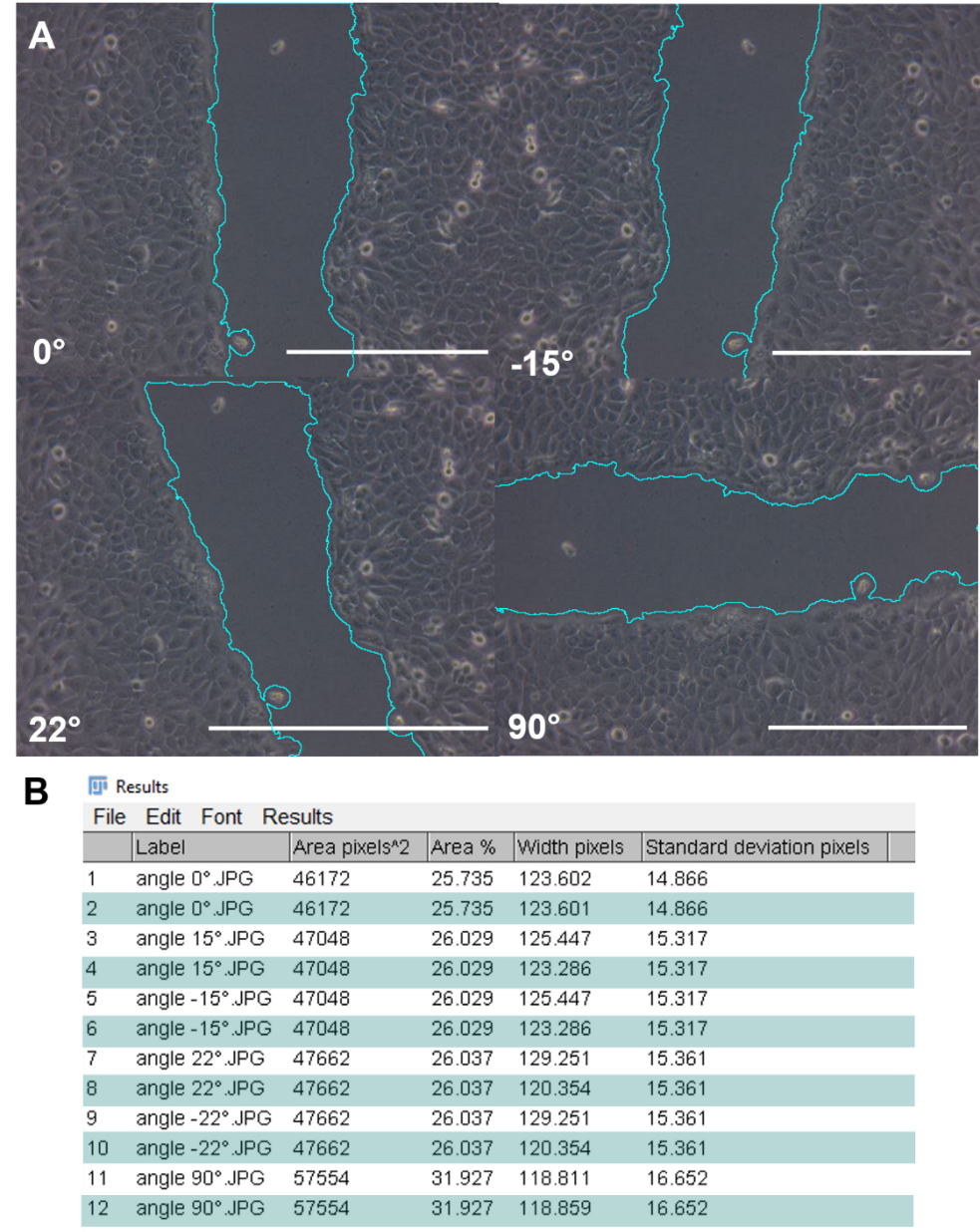


**S4 Fig. Fixing the width of the wound with respect to its inclination. A.** Wounds with different angles of inclination. Scale bar=200 pixels. B. Width measurements with (blue) and without correction(white).
