# Supplementary material for: An image J plugin for the high throughput image analysis of in vitro scratch wound healing assays
Source: PLoS One. 2020 Jul 28;15(7):e0232565. doi: 10.1371/journal.pone.0232565 (PMC7386569; doi:10.1371/journal.pone.0232565)
Supplement: S5 Fig — A. ROI selection in images analyzed with different set parameters B. List of parameters for the collected images. (DOCX) [file pone.0232565.s007.docx]

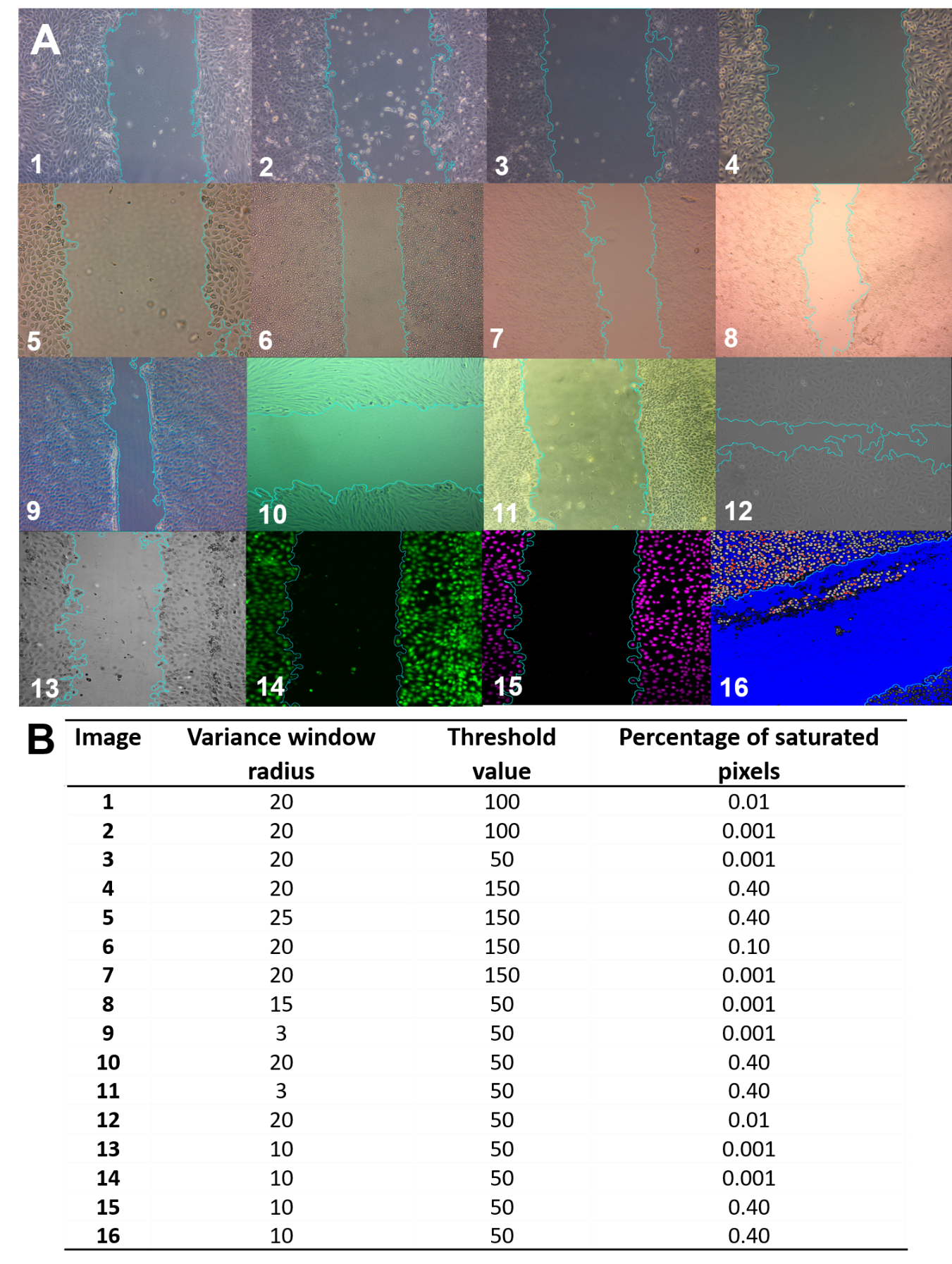
**S5 Fig. Images collected under different conditions. A.** ROI selection in images analyzed with different set parameters **B.** List of parameters for the collected images.
